# Comparison of emergency department and hospital admissions data for air pollution time-series studies

**DOI:** 10.1186/1476-069X-11-70

**Published:** 2012-09-21

**Authors:** A Winquist, M Klein, P Tolbert, WD Flanders, J Hess, SE Sarnat

**Affiliations:** 1 Department of Environmental Health, Rollins School of Public Health, Emory University, 1518 Clifton Road NE, Atlanta, GA, 30322, USA; 2 Department of Epidemiology, Rollins School of Public Health, Emory University, Atlanta, GA, USA; 3 Department of Biostatistics and Bioinformatics, Rollins School of Public Health, Emory University, Atlanta, GA, USA; 4 Department of Emergency Medicine, School of Medicine, Emory University, 1648 Pierce Drive NE, Atlanta, GA, 30322, USA

**Keywords:** Ambient air pollution, Hospital admissions, Emergency department visits, Time series analysis, Environmental epidemiology

## Abstract

**Background:**

Emergency department (ED) visit and hospital admissions (HA) data have been an indispensible resource for assessing acute morbidity impacts of air pollution. ED visits and HAs are types of health care visits with similarities, but also potentially important differences. Little previous information is available regarding the impact of health care visit type on observed acute air pollution-health associations from studies conducted for the same location, time period, outcome definitions and model specifications.

**Methods:**

As part of a broader study of air pollution and health in St. Louis, individual-level ED and HA data were obtained for a 6.5 year period for acute care hospitals in the eight Missouri counties of the St. Louis metropolitan area. Patient demographic characteristics and diagnostic code distributions were compared for four visit types including ED visits, HAs, HAs that came through the ED, and non-elective HAs. Time-series analyses of the relationship between daily ambient ozone and PM_2.5_ and selected cardiorespiratory outcomes were conducted for each visit type.

**Results:**

Our results indicate that, compared with ED patients, HA patients tended to be older, had evidence of greater severity for some outcomes, and had a different mix of specific outcomes. Consideration of ‘HA through ED’ appeared to more effectively select acute visits than consideration of ‘non-elective HA’. While outcomes with the strongest observed temporal associations with air pollutants tended to show strong associations for all visit types, we found some differences in observed associations for ED visits and HAs. For example, risk ratios for the respiratory disease-ozone association were 1.020 for ED visits and 1.004 for ‘HA through ED’; risk ratios for the asthma/wheeze-ozone association were 1.069 for ED visits and 1.106 for ‘HA through ED’. Several factors (e.g. age) were identified that may be responsible, in part, for the differences in observed associations.

**Conclusions:**

Demographic and diagnostic differences between visit types may lead to preference for one visit type over another for some questions and populations. The strengths of observed associations with air pollutants sometimes varied between different health care visit types, but the relative strengths of association generally were specific to the pollutant-outcome combination.

## Background

Many time-series studies have found associations between daily ambient air pollution levels and acute exacerbations of cardiovascular and respiratory diseases [1-3]. These studies commonly use the daily count of health care visits for selected conditions as the measure of morbidity in the population. Two widely considered types of visits are emergency department (ED) visits and hospital admissions (HAs). Data concerning both ED visits and HAs are routinely collected by hospitals for billing purposes and can be obtained from individual hospitals or, in some cases from centralized sources (e.g. Medicare, hospital associations or state health departments), without the need for costly data collection from individuals. Standardized variable fields [e.g., International Classification of Diseases, Ninth Revision, Clinical Modification (ICD-9) codes] are recorded for both ED visit and HA data [4-6].

However, important differences between ED visits and HAs may impact their usefulness for addressing particular questions, and the magnitude and interpretation of observed air pollution-health associations. Some of these differences simply reflect medical need for the different types of services offered in these two settings. For example, HAs are less frequent than ED visits; ED visits may represent generally less severe events than HAs; and ED visits require only action by the individual, while HAs also require action by a physician which could reduce subjectivity [7]. National surveys have also shown that the proportion of children among ED visits is higher than the proportion of children among HAs, while the reverse is true for patients aged 65 years or older [8]. Other differences between ED visits and HAs may not be related solely to medical need. Within a given geographic area and time period, access to primary care, ED care, and inpatient hospital care, and the way in which these services are used (as a result of patient or provider decisions), may differ in different sub-populations [9,10]. For example EDs may be used for primary care to a greater extent by those who, for economic reasons, have difficulty accessing primary care services [11,12].

An additional important difference between ED visits and HAs is that while ED visits are generally unscheduled, HAs for some types of outcomes are more frequently scheduled. In time-series studies, inclusion of scheduled admissions could attenuate observed associations with air pollution, due to inclusion of admissions for which timing of the event was not caused by air pollution. The approach to identifying and selecting unscheduled, or truly acute, visits has varied between studies. Some studies have not specifically restricted analyses to acute admissions [13-15], others have restricted analyses to admissions coded as non-elective, urgent, or emergency [16-19], and some have restricted analyses to admissions from the ED [20,21]. The approach to this issue could impact analytic results.

The choice of whether to consider one type of visit over the other may be determined based on the outcome or population of interest or may be dictated by data availability and cost. For investigators making decisions about types of visits to consider in air pollution time-series studies, little direct information is available regarding the potential impact of visit type and acute visit selection approaches on observed epidemiologic associations. Although many studies have considered one of the two types of visits, and these studies have been thoroughly reviewed [1,3], studies conducted in different time periods or different locations may not be directly comparable. Differences in results between studies using HAs and those using ED visits may be due to factors other than visit type, such as differences in pollution levels, populations, outcome definitions, or analytic models. Few studies have considered both ED visits and HAs in the same study, and in those studies that have considered both types, the time periods or outcome definitions have often differed, preventing direct comparison of results. Among studies that have included both visit types in a way that allows direct comparison, most have examined air pollution associations for asthma [22-25] or other respiratory outcomes [22,26] and few have examined associations for cardiovascular outcomes [26].

As part of a broader time-series study of air pollution and health in St. Louis, here we compare observed air pollution associations for ED visits and HAs for the same time period, geographic area, outcome definitions and model specifications. We also examine the extent to which demographic differences between patients and diagnostic differences between visit types might account for any differences in observed associations.

## Methods

Data were obtained from the Missouri Hospital Association for all ED visits and HAs to 28 of 29 acute care hospitals with emergency departments in the eight Missouri counties of the St. Louis metropolitan statistical area (MSA) during January 1, 2001 through June 27, 2007. Analyses included ED visits and HAs for patients residing in any one of 269 Zone Improvement Plan (ZIP) code areas located in the eight Missouri or eight Illinois counties of the St. Louis MSA. This study was approved by the Emory University Institutional Review Board.

ED visits were identified as all encounters designated as ED visits, as well as those coded as inpatient visits with either the admission source designated as the ED or with an indication of ED billing. HAs were identified as inpatient visits that were not the result of transfers from other hospitals, critical access hospitals or “other healthcare facilities.” Three separate datasets were created for analyses of HAs. The first included all HAs, without any selection of acute visits (referred to as ‘All HA’). The second included the subset of ED visits for which there was also an indication of HA (referred to as ‘HA through ED’). The third data set included the subset of HAs that had an admission type not categorized as elective (referred to as ‘non-elective HA’). The hospitals and the specific time periods included for each hospital were the same for all visit types.

Cardiorespiratory outcomes of interest were defined based on the primary ICD-9 diagnosis code for the visit, and included: a selected respiratory disease group (referred to as RD; ICD-9 codes 460–465, 466.0, 466.1, 466.11, 466.19, 477, 480–486, 491, 492, 493, 496, 786.07), pneumonia (ICD-9 codes 480–486), asthma or wheeze (ICD-9 codes 493, 786.07), a selected cardiovascular disease group (referred to as CVD; ICD-9 codes 410–414, 427, 428, 433–437, 440, 443–445, 451–453), dysrhythmia (ICD-9 code 427), and congestive heart failure (CHF; ICD-9 code 428) (see Additional file 1: Table S1 for a listing of the specific conditions included). Visits for the same condition on the same day were counted as a single visit. Asthma, pneumonia, dysrhythmia and CHF were selected a priori as specific diagnoses of interest because of their representation of different age groups, with asthma and dysrhythmia representing younger populations and pneumonia and CHF representing older populations.

The daily numbers of each visit type for each outcome were calculated overall and by sociodemographic characteristics, including age category (0–1 years, 2–18 years, 19–64 years and ≥65 years), and whether the patient’s residence was in one of 34 ZIP codes designated by Census 2000 as a poverty area (for which ≥20% of residents had incomes below the federal poverty level), which was used as a measure of socioeconomic status (SES). Method of payment recorded for the visit was also considered as a SES measure, but was found to be less useful due to its relationship with age (e.g. eligibility for Medicaid and Medicare varies with age).

Associations between daily counts for each visit type and daily ambient pollutant levels were examined using Poisson generalized linear models. Pollutants of interest included 8-hr maximum ozone and 24-hr average PM_2.5_ (particulate matter measuring ≤2.5 micrometers in diameter) using data from the US Environmental Protection Agency Air Quality System Tudor Street (site ID 171630010) and Blair Street (site ID 295100085) stations, respectively. To facilitate comparison of relationships with potentially different lag structures, we used distributed lag models [27], including lags of 0–4 days. Model specifications were selected based on models used in our previous air pollution time-series studies [28-30], as well as sensitivity analyses. The focus here was on comparison of results for different visit types using the same carefully selected model that was suitable for a range of outcomes. The final models controlled for time trends using cubic splines for day of visit with monthly knots, and indicator variables for day-of-week, holidays, season (in models for respiratory outcomes), and periods with different sets of hospitals with available data. Models also controlled for meteorology (using data from the National Climatic Data Center for St. Louis Lambert International Airport) including daily maximum temperature (lag 0) using indicator variables for each °C; daily minimum temperature (1–4 day moving average) using cubic terms; and mean dew point (0–2 day moving average) using cubic terms. Models were also fit that were stratified by age category or poverty area to determine whether differences in results for the different visit types were reduced when examined within sociodemographic strata. Risk ratios (RR) were expressed per interquartile range (IQR) change in pollutant concentrations.

## Results

### Characterization of visit types and outcomes

The final data sets included 5,709,926 ED visit records; 1,999,708 ‘all HA’ records; 1,024,228 ‘HA through ED’ records; and 1,401,619 ‘non-elective HA’ records. More than 90% of visits of each type were made by patients residing in the Missouri counties of the St. Louis MSA. For the RD and dysrhythmia outcomes, mean daily counts were highest for ED visits, followed by ‘All HA’, ‘non-elective HA’, and ‘HA through ED’ (Table 1). For the CVD and CHF outcomes, ‘All HA’ had the highest daily counts, followed by ED visits, ‘non-elective HA’, and ‘HA through ED’. The ratio of daily ED visit to ‘All HA’ counts varied by outcome, and was highest for RD (5.46) and lowest for CVD (0.85).

**Table 1:** Mean daily outcome counts by visit type, outcome, and selected characteristics, St. Louis, Missouri, 1/1/2001-6/27/2007

**Outcome**	**ICD-9 codes**	**Subgroup**	**ED Visits**	**All Hospital Admissions**		**Non-Elective Hospital Admissions**		**Hospital Admissions through ED**	
			**Mean Daily Count**	**Mean Daily Count**	**ED/HA ratio**	**Mean Daily Count**	**ED/HA ratio**	**Mean Daily Count**	**ED/HA ratio**
All Diagnoses	All	**Overall**	**2410.3**	**844.1**	**2.86**	**591.7**	**4.07**	**432.3**	**5.57**
		Age 0-1	146.49	94.06	1.56	91.48	1.60	10.38	14.11
		Age 2-18	519.02	42.66	12.17	34.42	15.08	25.55	20.32
		Age 19-64	1355.51	416.50	3.25	257.47	5.26	209.40	6.47
		Age ≥65	389.22	290.88	1.34	208.27	1.87	187.02	2.08
		Poverty area zip code	478.67	137.57	3.48	99.62	4.80	82.34	5.81
		Non-Poverty area zip code	1925.22	704.59	2.73	490.76	3.92	349.31	5.51
**Respiratory Outcomes**									
RD	460-465, 466.0, 466.1, 466.11, 466.19, 477, 480–486, 491, 492, 493, 496, 786.07	**Overall**	**259.6**	**47.6**	**5.46**	**41.5**	**6.25**	**39.3**	**6.61**
		Age 0-1	39.8	4.3	9.23	3.8	10.43	3.5	11.33
		Age 2-18	77.7	5.4	14.33	4.9	15.77	4.5	17.31
		Age 19-64	113.3	16.1	7.05	13.7	8.29	12.9	8.81
		Age ≥65	28.8	21.8	1.32	19.1	1.51	18.4	1.56
		Poverty area zip code	55.21	9.26	5.96	7.92	6.97	8.01	6.89
		Non-Poverty area zip code	203.49	38.23	5.32	33.56	6.06	31.19	6.52
Asthma/ Wheeze	493, 786.07	**Overall**	**46.9**	**8.7**	**5.40**	**7.8**	**6.02**	**7.4**	**6.31**
		Age 0-1	5.2	0.8	6.75	0.7	7.41	0.7	7.82
		Age 2-18	21.1	3.0	7.04	2.8	7.43	2.7	7.78
		Age 19-64	18.7	3.8	4.96	3.3	5.76	3.1	6.00
		Age ≥65	1.9	1.1	1.64	1.0	1.87	0.9	1.99
		Poverty area zip code	15.57	2.75	5.65	2.49	6.24	2.54	6.13
		Non-Poverty area zip code	31.20	5.91	5.28	5.28	5.91	4.88	6.40
Pneumonia	480-486	**Overall**	**41.4**	**25.2**	**1.64**	**21.9**	**1.89**	**20.9**	**1.98**
		Age 0-1	4.7	1.2	3.87	1.1	4.47	1.0	4.80
		Age 2-18	8.2	1.9	4.31	1.6	5.10	1.4	5.89
		Age 19-64	14.0	7.9	1.76	6.8	2.07	6.4	2.19
		Age ≥65	14.5	14.1	1.03	12.5	1.16	12.1	1.19
		Poverty area zip code	7.03	4.10	1.72	3.43	2.05	3.48	2.02
		Non-Poverty area zip code	34.32	21.06	1.63	18.49	1.86	17.39	1.97
**Cardiovascular Outcomes**									
CVD	410-414, 427, 428, 433–437, 440, 443–445, 451-453	**Overall**	**88.8**	**105.0**	**0.85**	**74.3**	**1.19**	**65.5**	**1.36**
		Age 0-1	0.2	0.1	2.07	0.1	2.65	0.1	3.25
		Age 2-18	0.4	0.2	2.68	0.1	3.59	0.1	4.60
		Age 19-64	34.1	38.1	0.90	25.9	1.32	22.4	1.53
		Age ≥65	54.0	66.7	0.81	48.2	1.12	42.9	1.26
		Poverty area zip code	14.65	15.47	0.95	11.42	1.28	10.81	1.36
		Non-Poverty area zip code	73.96	89.40	0.83	62.78	1.18	54.56	1.36
Dysrhythmia	427	**Overall**	**18.3**	**14.1**	**1.30**	**10.6**	**1.73**	**9.4**	**1.95**
		Age 0-1	0.1	0.0	3.63	0.0	4.14	0.0	4.92
		Age 2-18	0.3	0.1	4.06	0.1	6.05	0.0	7.47
		Age 19-64	7.8	4.6	1.71	3.4	2.28	3.0	2.56
		Age ≥65	10.1	9.4	1.07	7.1	1.42	6.3	1.60
		Poverty area zip code	2.72	1.73	1.57	1.32	2.06	1.26	2.17
		Non-Poverty area zip code	15.58	12.36	1.26	9.29	1.68	8.12	1.92
CHF	428	**Overall**	**22.4**	**24.2**	**0.92**	**20.1**	**1.11**	**18.7**	**1.20**
		Age 0-1	0.0	0.0	0.75	0.0	1.06	0.0	1.33
		Age 2-18	0.0	0.0	0.80	0.0	1.02	0.0	1.96
		Age 19-64	6.5	6.8	0.96	5.4	1.20	5.0	1.29
		Age ≥65	15.8	17.4	0.91	14.6	1.08	13.6	1.16
		Poverty area zip code	5.15	5.17	1.00	4.20	1.23	4.15	1.24
		Non-Poverty area zip code	17.20	19.04	0.90	15.91	1.08	14.54	1.18

*RD* respiratory disease group, *CVD* cardiovascular disease group, *CHF* congestive heart failure, *ED* emergency department, *HA* hospital admission, *ICD-9* international classification of diseases, 9th Revision, clinical modification.

#### Overlap between visit types

By definition, visits among the four visit types overlapped (Table 2). The percentage of ED visits that ultimately led to admission was much higher for CVD (73.8%) than for RD (15.1%). The percentage of ‘All HA’ that came through the ED also varied, being highest for respiratory outcomes (82.4%-85.9%) and lowest for cardiovascular outcomes (62.0%-76.9%). Across all outcomes, the percentage of visits that came through the ED was higher for ‘non-elective HA’ compared with ‘All HA’.

**Table 2:** Overlap of visits among the visit types, by outcome

**Case Group**	**Percentage of ED visits that were admitted**	**Percentage of hospital admissions that came through the ED**			**Percentage of visits coded as non-elective**			
		**All HA**	**Non-elective HA**	**HA through ED**	**ED**	**All HA**	**Non-elective HA**	**HA through ED**
All visits	17.9	50.8	69.8	100	89.9	70.1	100	96.3
RD	15.1	82.4	91.4	100	78.5	87.3	100	96.9
Asthma/Wheeze	15.8	85.9	93.0	100	95.9	89.8	100	97.2
Pneumonia	50.4	82.5	91.6	100	94.1	87.1	100	96.7
CVD	73.8	62.0	85.3	100	95.7	70.8	100	97.3
Dysrhythmia	51.2	66.3	85.9	100	97.2	75.3	100	97.6
CHF	83.7	76.9	89.7	100	96.8	83.0	100	96.8

*RD* respiratory disease group, *CVD* cardiovascular disease group, *CHF* congestive heart failure, *ED* emergency department, *HA* hospital admission.

The percentage of ‘All HA’ coded as non-elective was higher for respiratory outcomes (e.g., 87.3% for RD) than for cardiovascular outcomes (e.g., 70.8% for CVD). Some specific ICD-9 diagnosis codes had an especially high percentage of ‘All HA’ that were coded as elective [e.g., 414.01 (coronary atherosclerosis of native coronary artery) accounted for 21.2% of CVD ‘All HA’ (see Additional File 2: Table S2) and was elective for 51.6% of admissions]. Even some ED visits were coded as elective, primarily among respiratory visits (e.g., 21.5% for RD ED visits). For example, acute sinusitis (unspecified) (ICD-9 code 461.9) represented 6.9% of RD ED visits and was elective for 70.9% of visits; and acute bronchitis (ICD-9 code 466.0) represented 8.6% of RD ED visits and was elective for 37.3% of visits. However, for ED visits subsequently admitted to the hospital, the non-elective percentage was uniformly high across all outcomes (>96%; Table 2).

#### Comparison of diagnostic composition of outcome groups

The diagnoses represented within the outcome groups varied between visit types (see Additional file 2: Table S2). For some outcomes, ICD-9 code distributions indicated more severe outcomes among HAs compared with ED visits. For example, for asthma/wheeze, the percentage of patients with primary ICD codes of 493.01 and 493.91 (indicating status asthmaticus) was substantially higher among HAs (~30%) compared with ED visits (~13%). In other cases, the diagnostic differences indicated different relative representation of specific conditions within the outcome groups. For example in the RD group, pneumonia was substantially more common among HAs than ED visits. For dysrhythmia, ICD-9 codes 427.1, 427.31 and 427.81 (paroxysmal ventricular tachycardia, atrial fibrillation and sinoatrial node dysfunction) were more common among HAs than ED visits; and ICD-9 codes 427.0 and 427.5 (paroxysmal supraventricular tachycardia and cardiac arrest) were more common among ED visits than among HAs. For CVD, ICD-9 code 414.01 (coronary atherosclerosis of native coronary artery) was most common for ‘All HAs’.

#### Demographic comparisons

There was higher representation of younger age groups among ED visits than among HAs, as expected (Table 1). For example, for respiratory visits, the proportion of patients aged ≤18 years was higher among ED visits than HAs (e.g., 45.3% of RD ED vs. 20.4% of RD ‘All HA’). In addition, the proportion of people aged 65 and older was higher among all HAs than among ED visits (e.g., 11.1% of RD ED vs. 45.8% of RD ‘All HA’), particularly for respiratory and dysrhythmia outcomes.

The percentage of visits for any outcome that were made by patients residing in poverty area ZIP codes was slightly higher among ED visits (19.9%) and ‘HA through ED’ (19.0%) than among ‘All HA’ (16.3%) and ‘Non-elective HA’ (16.8%). ED/HA ratios were also slightly higher for visits made by patients residing in poverty area compared to non-poverty area ZIP codes for all outcomes (Table 1), indicating slightly higher representation of people from poverty areas among ED visits than among HAs.

#### Temporal patterns

**Figure 1:**
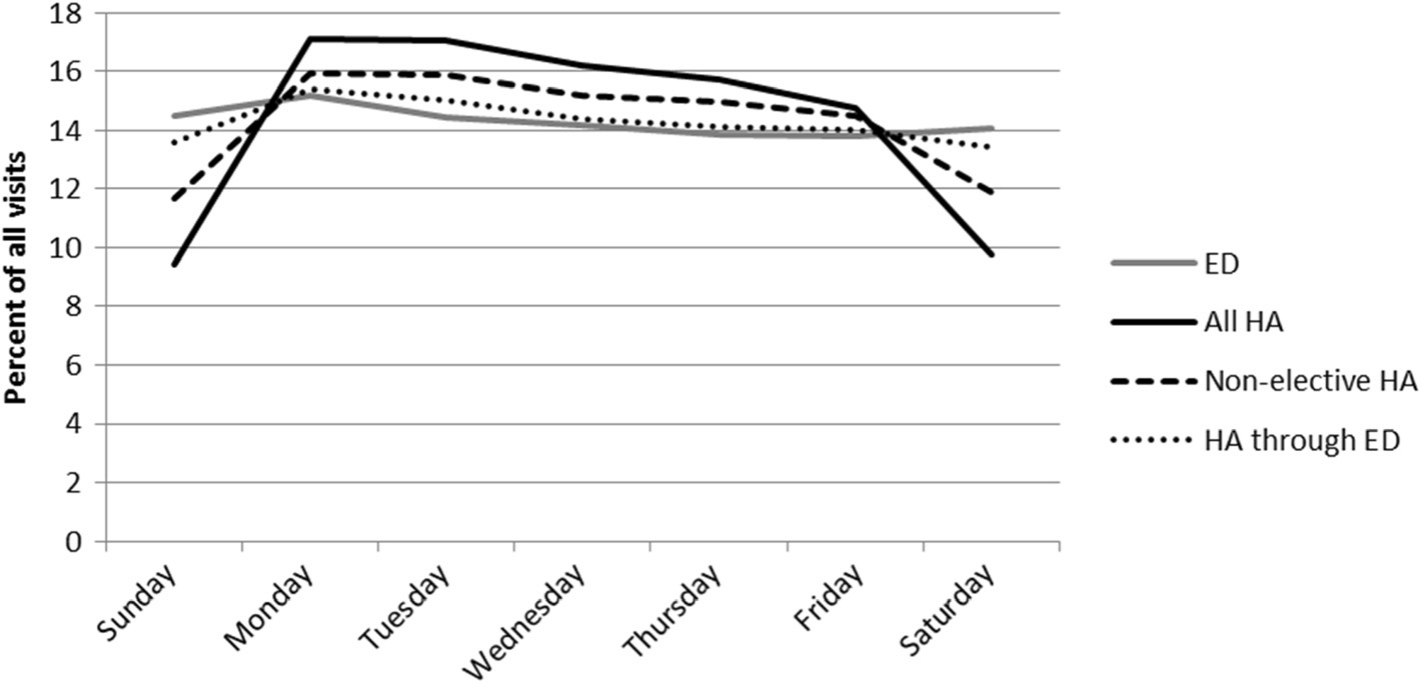
Overall percentage of visits by day of week among visits of each type.

ED visits generally showed the least variation in mean visit counts across days of the week (Figure 1). Progressively more variation across days of the week was seen for ‘HA through ED’, ‘non-elective HA’, and ‘All HA’. HAs showed lowest counts on the weekends and highest counts on weekdays, especially Monday, regardless of the outcome (data not shown). For ED visits, the weekly pattern differed by outcome, with respiratory ED visit counts highest on weekends and cardiovascular ED visit counts highest on weekdays, but less markedly than for HAs.

Correlations of the daily counts among the HA subsets were high (Spearman correlation coefficient (r) > 0.7), especially for respiratory outcomes (r > 0.93) (Table 3). Correlations of daily counts between ED visits and the HA subsets were generally lower, but were highest for ‘HA through ED’ (r = 0.63 for all visits), as might be expected given that ‘HA through ED’ were a subset of ED visits.

**Table 3:** Spearman correlations between daily outcome counts for each visit type

**Outcome**		**All ED**	**All HA**	**Non-elective HA**
All visits	All HA	0.35		
	Non-elective HA	0.38	0.94	
	HA through ED	0.63	0.73	0.78
RD	All HA	0.72		
	Non-elective HA	0.75	0.97	
	HA through ED	0.77	0.93	0.97
Asthma/Wheeze	All HA	0.62		
	Non-elective HA	0.63	0.96	
	HA through ED	0.63	0.93	0.96
Pneumonia	All HA	0.81		
	Non-elective HA	0.83	0.96	
	HA through ED	0.85	0.93	0.96
CVD	All HA	0.73		
	Non-elective HA	0.83	0.89	
	HA through ED	0.90	0.71	0.85
Dysrhythmia	All HA	0.58		
	Non-elective HA	0.68	0.85	
	HA through ED	0.74	0.72	0.89
CHF	All HA	0.78		
	Non-elective HA	0.87	0.91	
	HA through ED	0.92	0.82	0.92

*RD* respiratory disease group, *CVD* cardiovascular disease group, *CHF* congestive heart failure, *ED* emergency department, *HA* hospital admission.

### Air quality data

During the study period (1/2001-6/2007), 8-hr maximum ozone measurements were available for 2,323 days (missing on 46 days, 1.9%), with an average concentration of 36.3 parts per billion (ppb) (standard deviation = 18.6 ppb, range = 1.0-111.8 ppb, IQR = 27.3 ppb). Measurements of 24-hour average PM_2.5_ were available for 2,300 days (missing on 69 days, 2.9%), with an average concentration of 14.4 micrograms per cubic meter (μg/m^3^) (standard deviation = 7.5μg/m^3^, range = 0.4-56.6 μg/m^3^, IQR = 9.3 μg/m^3^). The Pearson correlation coefficient between the daily 8-hr ozone and 24-hr PM_2.5_ measurements was 0.25.

### Epidemiologic results

#### Main results

**Figure 2:**
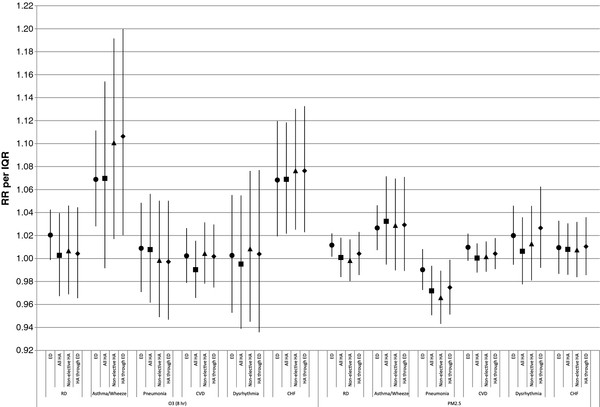
**Associations between cardiorespiratory outcomes and O**_**3**_**and PM**_**2.5**_**by visit type.** Displayed risk ratios are the exponentiated sum of the coefficients for lags 0–4 from distributed lag models. Risk ratios are computed per interquartile range of pollutant concentrations (27.3 ppb for 8-hr maximum O_3_; 9.3 μg/m^3^ for 24-hr average PM_2.5_). RR: risk ratio, IQR: interquartile range, O_3_: ozone, PM_2.5_: particulate matter ≤2.5 micrometers in diameter, RD: respiratory disease group, CVD: cardiovascular disease group, CHF: congestive heart failure, ED: emergency department, HA: hospital admission.

Results of time-series models for the associations between daily cardiorespiratory outcome counts and daily ozone and PM_2.5_ concentrations are shown in Figure 2 (and in tabular form in Additional file 3: Table S3 and Additional file 4: Table S4). Overall, the asthma-ozone and CHF-ozone associations were the strongest observed, and these strong associations were relatively consistent across visit types. When comparing across visit types, the visit type for which the strongest association was observed varied by pollutant-outcome combination. For example, for RD, associations were stronger for ED visits than for HAs (e.g., for ozone: RR using ED visits = 1.020, 95% confidence interval (CI) =0.999-1.043; RR using ‘All HA’ = 1.003, 95% CI = 0.967-1.039). For asthma/wheeze-ozone, however, the association was strongest for ‘HA through ED’ (RR = 1.106, 95% CI = 1.020-1.200) and ‘non-elective HA’ (RR = 1.101, 95% CI = 1.017-1.192), but was weaker for ED visits (RR = 1.069, 95% CI = 1.028-1.111) and ‘All HA’ (RR = 1.070, 95% CI = 0.992-1.154).

For respiratory outcomes, the standard errors of the risk ratio estimates were smaller for ED visits than for HAs, due to the differences in the mean daily counts. This difference was less pronounced for cardiovascular outcomes for which the mean daily counts were more similar. The statistical significance of associations between the pollutants and outcomes was different among the visit types in some cases. For example, the RD-PM_2.5_ and asthma/wheeze- PM_2.5_ associations were statistically significant only for ED visits.

#### Results by age and poverty area

**Figure 3:**
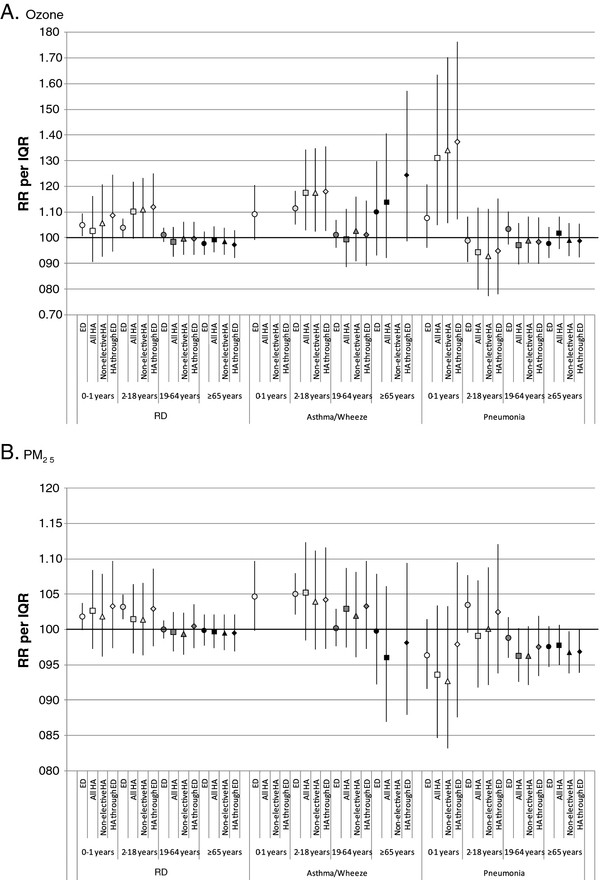
**Associations between respiratory outcomes and O**_**3**_**(A) and PM**_**2.5**_**(B) by visit type and age category.** Displayed risk ratios are the exponentiated sum of the coefficients for lags 0–4 from distributed lag models. Risk ratios are computed per interquartile range of pollutant concentrations (27.3 ppb for 8-hr maximum O_3_; 9.3 μg/m^3^ for 24-hr average PM_2.5_). RR: risk ratio, IQR: interquartile range, O_3_: ozone, PM_2.5_: particulate matter ≤2.5 micrometers in diameter, RD: respiratory disease group, CVD: cardiovascular disease group, CHF: congestive heart failure, ED: emergency department, HA: hospital admission.

**Figure 4:**
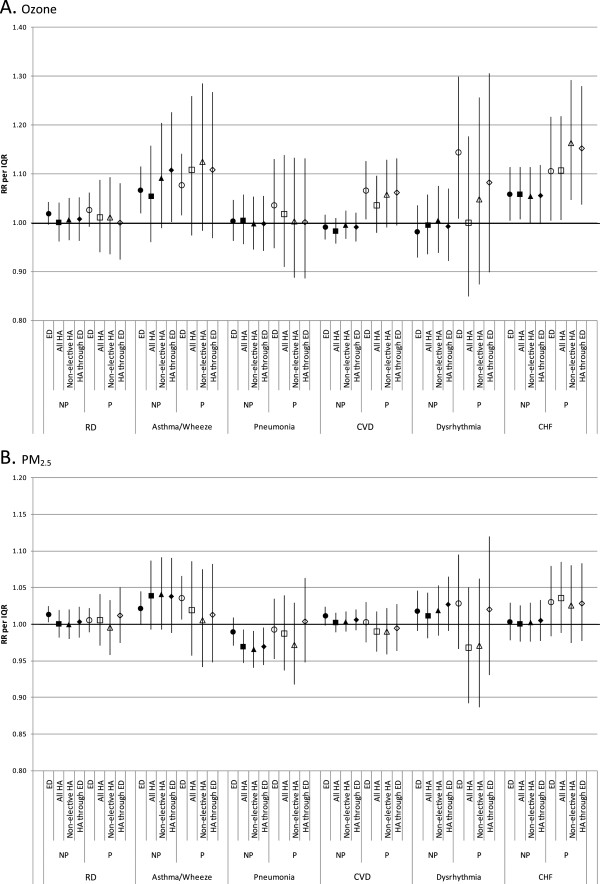
**Associations between cardiorespiratory outcomes and O3 (A) and PM2.5 (B) by visit type and poverty area.** Displayed risk ratios are the exponentiated sum of the coefficients for lags 0–4 from distributed lag models. Risk ratios are computed per interquartile range of pollutant concentrations (27.3 ppb for 8-hr maximum O_3_; 9.3 μg/m^3^ for 24-hr average PM_2.5_). RR: risk ratio, IQR: interquartile range, O_3_: ozone, PM_2.5_: particulate matter ≤2.5 micrometers in diameter, P: poverty area, NP: non-poverty area, RD: respiratory disease group, CVD: cardiovascular disease group, CHF: congestive heart failure, ED: emergency department, HA: hospital admission.

We considered models stratified by age and by poverty area (Figures 3 and 4, in tabular form in Additional File 3: Table S3 and Additional File 4: Table S4) to examine whether these factors explained some of the variation in associations by visit type (e.g., if stratum-specific effect estimates are more similar than overall results).

For both ozone and PM_2.5_, associations for the RD outcome were generally stronger in younger age groups (0–1 years and 2–18 years) than in older age groups for all visit types. In the younger age groups for RD, magnitudes of association were higher for ‘HA through ED’ than for ED visits, whereas the association was strongest for ED visits in the overall analysis (Figure 2). For the pneumonia-ozone relationship, the associations were strongest in the 0–1 year age group, and in that age group the association was stronger for HAs (especially ‘HA through ED’ for which RR = 1.374, 95% CI = 1.071-1.763) than for ED visits (RR = 1.076, 95% CI = 0.960-1.207) but the estimates were imprecise. For the asthma/wheeze-ozone relationship, observed associations were strongest in the 2–18 year age group, for which the association was also stronger for HAs than for ED visits. For cardiovascular outcomes, the number of visits in the various age groups permitted stratification only for the 19–64 year and ≥65 year age groups; differences in observed associations between the visit types within each age group largely paralleled differences observed overall (see Additional file 3: Table S3 and Additional file 4: Table S4).

When models were stratified by poverty area, some overall differences in epidemiologic associations were observed. Most notably, the CVD-ozone and CHF-ozone associations were uniformly stronger for patients from poverty areas than non-poverty areas, regardless of visit type. However, the differences in associations across visit types were sometimes more pronounced within poverty-area strata than in the overall analysis, rather than associations being more consistent across visit types within strata. For the dysrhythmia outcome, the association with ozone was stronger for ED visits than for HAs in poverty areas, but this difference was not seen in non-poverty areas. Stratification by both age and poverty area was generally not possible due to insufficient daily visit counts.

## Discussion

The overarching goals of our study were to identify and characterize differences between ED visits and HAs that may be important to consider in air pollution health effects studies, and to illustrate the potential impact of these differences on analytic results. Our results illustrate the kinds and possible magnitude of these differences. In particular, as previous studies have found, patients visiting the ED were younger than those being hospitalized. Patients with ED visits or HAs through the ED were more likely to be from poor areas compared with all patients who were hospitalized. Hospitalized patients tended to have outcomes of greater severity than patients visiting the ED for some disease classes (as was observed for asthma). Some severe outcomes that are rapidly fatal (such as cardiac arrest) were more common among ED visits than among HAs. Overall, hospitalized patients simply have a different mix of disease outcomes compared with ED patients.

Our results also illustrate differences that can be observed for air pollution-health associations estimated using ED visits and HAs. While the outcomes with strongest temporal associations with air pollutants tended to show strong associations for all visit types, there were some notable differences in observed associations between visit types. The estimated associations for some outcomes were stronger when based on ED visits than when based on HAs (e.g., for RD-ozone), yet the opposite was true for other outcomes (e.g., for asthma/wheeze-ozone). Our results also indicate that power differences may be important to consider when selecting a visit type or when comparing results of analyses considering different visit types. Mean daily ED visit counts were higher than mean daily HA counts for many outcomes, which may yield greater analytic power for analyses considering ED visits than for those considering HAs, particularly for respiratory outcomes. This, in turn, may affect conclusions based on statistical significance. Differences between visit types in mean daily counts were not as pronounced for cardiovascular outcomes, so power considerations may be less important when comparing analyses using ED visits and HAs for cardiovascular outcomes.

Our results also suggest ways in which the descriptive differences between visit types may help us understand observed differences in epidemiologic associations across visit types. Different observed associations may partially reflect underlying differences in age, socioeconomic status and illness severity between the visit types if the concentration-response pattern differs according to these characteristics. We observed some evidence for this impacting our results. For example, differences in RD-ozone associations between visit types appeared to be related, in part, to differential age composition and diagnoses. There was greater representation of younger age groups among ED visits than among HAs, and for the RD-ozone relationship, observed associations were strongest in the young. Therefore, the apparent greater strength of the RD-ozone association when using ED visits may have been due to the higher proportion of younger patients among ED visits. In addition, RD HAs included a higher proportion of pneumonia visits, which had only a weak association with ozone overall. This could also contribute to the stronger RD-ozone association observed for ED visits, which may have included a higher proportion of outcomes with a stronger association with ozone. Differences in SES could also impact observed associations. However, consideration of the potential impact of SES differences between the visit types was difficult because the amount of SES information available was limited and the differences between visit types in the proportion of patients from poverty areas were less pronounced than age differences. Factors other than those considered here, as well as chance, could also explain the differences in the observed associations between the various visit types.

In addition to acute visits, HAs include non-acute and scheduled visits for which the timing of the visit is unlikely to be caused by air pollution. The timing of non-acute visits may be influenced by convenience, hospital workload and staffing, and other factors unrelated to the timing of disease exacerbations. The two approaches that we examined for selection of acute admissions each have strengths and weaknesses. Consideration of ‘HA through ED’ appears to have more effectively selected truly acute visits than consideration of ‘non-elective HA’. Assuming that ED visits are nearly all acute, some factors supporting this observation include: 1) there were often higher correlations between ‘HA through ED’ and ED visits than between ‘non-elective HA’ and ED visits, 2) the pattern across days of the week was closer to that observed for ED visits for ‘HA through ED’ than for ‘non-elective HA’, 3) among ‘HA through ED’ there was a uniformly high percentage of visits coded as non-elective, while among ‘non-elective HA’ the percentage of admissions that came through ED was not as high, especially for cardiovascular outcomes, and 4) for one diagnosis with a very high percentage coded as elective (i.e., ICD-9 code 414.01), the percentage of the overall CVD case group that it represented was more similar between ED visits and ‘HA through ED’ than between ED visits and ‘non-elective HA’. In addition, there was evidence of some inconsistency in how visits were coded as non-elective, including apparent changes in coding practices at some hospitals, which could impact results of analyses using the elective classification for visit selection. Furthermore, the designation of a visit as elective may not always reflect the acute vs. non-acute nature of the condition onset but rather the urgency of the medical need, as evidenced by the fact that a substantial proportion of ED visits for some conditions were coded as elective. The definition of an elective visit according to the Centers for Medicare and Medicaid Services instructions for the UB-04 form is “the patient’s condition permitted adequate time to schedule the availability of a suitable accommodation.” [31] Nevertheless, consideration of ‘HA through ED’ may also have disadvantages. Consideration of only ‘HA through ED’ may exclude some truly acute direct admissions by selecting only visits originating in the ED. In addition, the daily counts for ‘HA through ED’ were lower than those for the other visit types, potentially reducing power.

Some previous air pollution time series studies have included separate analyses of ED visits and HAs for the same location, but the results of analyses considering the two visit types are not always directly comparable due to differences in outcomes considered, time periods, age groups, or measures of association, or to insufficient information for comparison of the observed magnitude of effects [16,32-36]. A limited number of previous studies have considered both ED visits and HAs in a way that allows direct comparison of epidemiologic results for PM_2.5_ or ozone. While none focused attention on such a comparison, there are similarities between these previous results and the current findings. Slaughter, et al. [22] examined associations between PM and ED visits and HAs (‘All HA’) for respiratory conditions in Spokane, Washington. They noted a higher number of respiratory ED visits than respiratory HAs. For analyses of PM_2.5_, their results were similar to ours, in that the associations between all-respiratory visits and PM_2.5_ were slightly stronger for ED visits than for HAs (for all ages combined). In a study conducted in Washington, D.C., Babin, et al. [23] found a stronger association between asthma and ozone in the 5–17 year age group for ‘HA through ED’ than for ED visits, although associations were significant regardless of the visit type.

Our analyses had several limitations. Use of administrative data has limitations [5,6,37-39] which apply to both HA and ED visit data. SES was examined at the ZIP-code level, which is not ideal. In addition, we had no measure of severity of illness for most outcomes. While our model specifications were carefully selected and based on our previous time-series studies, we recognize that all models have some degree of misspecification. To focus on the visit-type comparison, we felt that it was important to use the same model specifications for all visit types. Finally, the generalizability of our specific findings may be limited. Since health care usage patterns, health care access, practice patterns among providers (for example, the extent of use of admissions for observation), and population composition can change over time and between locations, the specific differences between the types of health care visits and the associated differences in the results of analyses may not be the same in other time periods and other locations.

The results of the stratified analyses should be interpreted with caution. The purpose of the stratified analyses in this study was to determine whether differences in air pollution-outcome associations between the different types of health care visits could potentially be explained by demographic differences. However, some of the stratified analyses were based on small daily counts, resulting in very wide confidence intervals. In addition, it must be recognized that observed differences between demographic groups in the strength of the associations could be due to many factors, such as truly different effects of air pollution in different groups, different levels of exposure measurement error, or chance. Different levels of exposure measurement error may be present for different groups defined by geography when using central site monitoring data to assess exposures, however high correlations (r > 0.92) for ozone and PM_2.5_ between the sites used in the current analysis and other sites in the study area limit this concern.

## Conclusions

The findings of this study have several implications for future air pollution research. First, the demographic and diagnostic differences between the different types of health care visits may lead to one type of visit being preferred over another to study certain questions. For example, use of ED visits might be preferred over use of HAs to study outcomes in young populations, milder respiratory outcomes, or rapidly fatal outcomes such as cardiac arrest. Conversely, HAs might be preferred for studies of older populations. The choice of the type of visit considered may have a smaller impact on the results of studies of older populations and many cardiovascular outcomes. Broad diagnostic categories like all-respiratory diseases and all-cardiovascular diseases may have different compositions among visits of different types, and such differences should be taken into account when comparing results of studies considering different types of health care visits. It appears that consideration of HAs through the ED may be a more effective strategy for selecting acute HAs than consideration of non-elective HAs, but this must be balanced against the potential loss of power that may accompany this strategy. Finally, while the outcomes with the strongest temporal associations with air pollutants tended to show strong associations for all visit types, the strengths of the associations sometimes varied between visit types, with the relative strengths of association being specific to the pollutant-outcome combination. Overall, these results can help inform visit type selection decisions in the design of future studies, as well as the interpretation and comparison of studies using different visit types.

## Abbreviations

CHF: Congestive heart failure (ICD-9 code 428); CI: Confidence interval; CVD: An ‘all cardiovascular disease’ category defined as ICD-9 codes 410–414 427, 428, 433–437, 440, 443–445, 451–453; ED: Emergency department; HA: Hospital admission; ICD-9: International Classification of Diseases 9th Revision, Clinical Modification; IQR: Interquartile range; MSA: Metropolitan area; O_3_: Ozone; PM_2.5_: Particulate matter ≤2.5 micrometers in diameter; Ppb: Parts per billion; R: Correlation coefficient; RD: An ‘all respiratory disease’ category defined as ICD-9 codes 460–465 466.0, 466.1, 466.11, 466.19, 477, 480–486, 491, 492, 493, 496, 786.07; RR: Risk ratio; SES: Socioeconomic status; μg/m^3^: Micrograms per cubic meter; ZIP codes: United States postal Service Zone Improvement Plan codes.

## Competing interests

The authors declare that they have no competing interests.

## Authors’ contributions

AW participated in the design of the study, conducted data analysis, participated in interpretation of the findings, and drafted the manuscript. MK participated in the design of the study, provided frequent consultation on data analysis and participated in interpretation of the findings. PT and WDF participated in the design of the study, provided consultation on data analysis and participated in interpretation of the findings. JH provided consultation on interpretation of the findings. SES acquired the data, participated in the design of the study, provided frequent consultation on data analysis and participated in interpretation of the findings. All authors critically reviewed and approved the manuscript.

## Supplementary Material

Additional file 1 **Table S1. **Cardiorespiratory outcomes of interest, defined based on the primary ICD-9 diagnosis code for the visit. Description: Table listing specific conditions included in outcome groups. Click here for file

Additional file 2 **Table S2.** Percentage of records in each case group that had specific Primary ICD-9 codes, by visit type overall and by age group. Description: Table listing the predominant diagnoses represented within the various outcome groups, and the percentage of the outcome group represented by each of those diagnoses. Click here for file

Additional file 3 **Table S3.** Summary (lags 0–4) distributed lag model risk ratios for each data set for Ozone, Tudor Street monitor, RR expressed per IQR (27.3 ppb) increment. Description: Table of results of overall time series models for the associations between daily counts of visits for the selected outcomes and daily ozone concentrations. Click here for file

Additional file 4 **Table S4.** Summary (lags 0–4) distributed lag model risk ratios for each data set for PM_2.5_, Blair Street Monitor, RR expressed per IQR (9.3 μg/m^3^) increment. Description: Table of results of overall time series models for the associations between daily counts of visits for the selected outcomes and daily PM_2.5_ concentrations. Click here for file
