# Supplementary material for: Comparison of emergency department and hospital admissions data for air pollution time-series studies
Source: Environ Health. 2012 Sep 21;11:70. doi: 10.1186/1476-069X-11-70 (PMC3511882; doi:10.1186/1476-069X-11-70)
Supplement: Additional file 1 — Table S1. Cardiorespiratory outcomes of interest, defined based on the primary ICD-9 diagnosis code for the visit. Description: Table listing specific conditions included in outcome groups. [file 1476-069X-11-70-S1.pdf]

Table A.1. Cardiorespiratory outcomes of interest, defined based on the primary ICD-9 diagnosis code for the visit

Case Group	ICD-9-CM codes included	Description of Disease Entity
**RD**	460-465, 466.0, 466.1, 466.11, 466.19, 477, 480-486, 491, 492, 493, 496, 786.07	Acute nasopharyngitis [common cold]; Acute sinusitis; Acute pharyngitis; Acute tonsillitis; Acute laryngitis and tracheitis; Acute upper respiratory infections of multiple or unspecified sites; Acute bronchitis; Acute bronchiolitis; Acute bronchiolitis due to respiratory syncytial virus (RSV); Acute bronchiolitis due to other infectious organisms; Allergic rhinitis; Viral pneumonia; Pneumococcal pneumonia [Streptococcus pneumoniae pneumonia]; Other bacterial pneumonia; Pneumonia due to other specified organism; Pneumonia in infectious diseases classified elsewhere; Bronchopneumonia, organism unspecified; Pneumonia, organism unspecified; Chronic bronchitis; Emphysema; Asthma; Chronic airway obstruction, not elsewhere classified; Wheezing
**Pneumonia**	480-486	Viral pneumonia; Pneumococcal pneumonia [Streptococcus pneumoniae pneumonia]; Other bacterial pneumonia; Pneumonia due to other specified organism; Pneumonia in infectious diseases classified elsewhere; Bronchopneumonia, organism unspecified; Pneumonia, organism unspecified;
**Asthma or Wheeze**	493, 786.07	Asthma; Wheezing
**CVD**	410-414, 427, 428, 433-437, 440, 443-445, 451-453	Acute myocardial infarction; Other acute and subacute forms of ischemic heart disease; Old myocardial infarction; Angina pectoris; Other forms of chronic ischemic heart disease; Cardiac dysrhythmias; Heart failure; Occlusion and stenosis of precerebral arteries; Occlusion of cerebral arteries; Transient cerebral ischemia; Acute, but ill-defined, cerebrovascular disease; Other and ill-defined cerebrovascular disease; Atherosclerosis; Other peripheral vascular disease; Arterial embolism and thrombosis; Atheroembolism; Phlebitis and thrombophlebitis; Portal vein thrombosis; Other venous embolism and thrombosis
**Dysrhythmia**	427	Cardiac dysrhythmias
**CHF**	428	Heart failure

ICD-9: International Classification of Diseases, 9th Revision, Clinical Modification, RD: respiratory disease group, CVD: cardiovascular disease group, CHF: congestive heart failure
