# Supplementary material for: Comparison of emergency department and hospital admissions data for air pollution time-series studies
Source: Environ Health. 2012 Sep 21;11:70. doi: 10.1186/1476-069X-11-70 (PMC3511882; doi:10.1186/1476-069X-11-70)
Supplement: Additional file 2 — Table S2. Percentage of records in each case group that had specific Primary ICD-9 codes, by visit type overall and by age group. Description: Table listing the predominant diagnoses represented within the various outcome groups, and the percentage of the outcome group represented by each of those diagnoses. [file 1476-069X-11-70-S2.pdf]

Table A.2. Percentage of records in each case group that had specific Primary ICD-9 codes, by visit type overall and by age group.*

ICD-9 Code	Interpretation	Overall				Age 0-1 years				Age 2-18 years				Age 19-64 years				Age ≥65 years			
		ED	All HA	Non-elective HA	HA through ED	ED	All HA	Non-elective HA	HA through ED	ED	All HA	Non-elective HA	HA through ED	ED	All HA	Non-elective HA	HA through ED	ED	All HA	Non-elective HA	HA through ED
RD (Overall N)		***614944***	***112676***	***98405***	***92999***	***94203***	***10202***	***9034***	***8311***	***184092***	***12846***	***11671***	***10633***	***268446***	***38067***	***32394***	***30467***	***68201***	***51560***	***45305***	***43587***
461.9	Acute sinusitis, unspecified site (%)	6.85	0.17	0.15	0.14	0.99	0.04	0.04	0.05	3.89	0.18	0.15	0.13	12.23	0.30	0.29	0.25	1.71	0.09	0.08	0.08
462	Acute pharyngitis (%)	14.87	0.36	0.36	0.34	3.82	0.14	0.14	0.16	22.09	0.72	0.74	0.67	17.10	0.71	0.71	0.70	1.83	0.04	0.05	0.05
464.4	Croup (%)	2.45	0.40	0.42	0.43	7.16	2.97	3.07	3.32	4.51	1.12	1.17	1.19	0.01	0.00	0.00	0.00	0.00	0.00	0.00	0.00
465.9	Acute upper respiratory infection, unspecified site (%)	19.81	1.40	1.42	1.41	42.30	5.73	6.27	6.24	20.89	2.06	2.16	2.10	15.04	1.19	1.06	1.10	4.59	0.54	0.52	0.53
466.0	Acute bronchitis (%)	8.56	2.33	2.31	2.27	1.58	0.51	0.45	0.40	3.93	0.36	0.34	0.33	14.35	3.34	3.36	3.26	7.95	2.44	2.43	2.40
466.11	Acute bronchiolitis due to respiratory syncytial virus (RSV) (%)	1.05	2.97	3.04	2.90	6.39	30.27	30.42	29.94	0.23	1.99	2.04	1.99	0.00	0.00	0.00	0.00	0.00	0.00	0.00	0.00
466.19	Acute bronchiolitis due to other infectious organisms (%)	1.88	1.32	1.28	1.21	11.27	13.52	12.92	12.48	0.42	0.68	0.62	0.63	0.06	0.05	0.04	0.04	0.03	0.02	0.01	0.01
486	Pneumonia, organism unspecified (%)	14.37	45.10	45.05	45.66	10.34	20.11	19.84	20.14	9.62	28.16	26.03	25.51	11.35	42.66	42.81	43.01	44.60	56.07	56.57	57.28
491.21	Obstructive chronic bronchitis, with (acute) exacerbation (%)	3.73	15.40	15.19	15.23	0.01	0.01	0.01	0.01	0.02	0.03	0.03	0.01	3.43	15.85	15.73	15.73	20.09	21.94	21.73	21.51
493.01	Extrinsic asthma, with status asthmaticus (%)	0.15	0.71	0.78	0.85	0.07	0.51	0.55	0.67	0.43	5.36	5.77	6.48	0.02	0.14	0.15	0.13	0.00	0.00	0.00	0.00
493.02	Extrinsic asthma, with (acute) exacerbation (%)	1.57	1.52	1.60	1.67	1.54	2.45	2.59	2.82	4.13	8.26	8.57	9.40	0.21	0.99	1.01	1.01	0.02	0.04	0.03	0.03
493.22	Chronic obstructive asthma, with (acute) exacerbation (%)	0.67	3.49	3.52	1.79	0.00	0.01	0.00	0.00	0.01	0.05	0.05	0.05	0.93	6.11	6.24	6.32	2.33	3.10	3.17	3.12
493.90	Asthma, unspecified type, unspecified exacerbation status (%)	4.95	0.93	0.86	0.81	3.20	1.62	1.51	1.38	6.79	1.50	1.39	1.34	5.21	1.36	1.25	1.21	1.34	0.33	0.31	0.29
493.91	Asthma, unspecified type, with status asthmaticus (%)	0.83	3.96	4.40	4.43	0.73	6.24	6.85	7.22	1.97	25.76	27.97	29.17	0.27	1.28	1.29	1.29	0.05	0.06	0.06	0.05
493.92	Asthma, unspecified, with (acute) exacerbation (%)	8.06	6.58	6.53	6.56	3.34	4.17	4.13	3.95	10.73	11.40	10.93	10.78	9.37	12.76	12.97	13.41	2.23	1.29	1.26	1.24
Asthma/Wheeze (Overall N)		***111000***	***20537***	***18436***	***17584***	***12236***	***1812***	***1651***	***1565***	***49978***	***7095***	***6724***	***6426***	***44400***	***8955***	***7711***	***7394***	***4386***	***2675***	***2350***	***2199***
493.00	Extrinsic asthma, unspecified exacerbation status (%)	4.53	0.67	0.67	0.64	13.44	3.26	3.33	3.32	6.16	0.75	0.73	0.67	0.66	0.20	0.19	0.18	0.32	0.30	0.21	0.18
493.01	Extrinsic asthma, with status asthmaticus (%)	0.82	3.88	4.18	4.47	0.51	2.87	3.03	3.58	1.60	9.70	10.01	10.72	0.11	0.61	0.61	0.55	0.00	0.04	0.00	0.00
493.02	Extrinsic asthma, with (acute) exacerbation (%)	8.72	8.32	8.55	8.86	11.96	13.80	14.17	14.95	15.29	14.95	14.87	15.59	1.26	4.20	4.25	4.17	0.39	0.79	0.60	0.64
493.22	Chronic obstructive asthma, with (acute) exacerbation (%)	3.72	19.15	18.79	18.77	0.01	0.06	0.00	0.00	0.03	0.10	0.09	0.08	5.66	25.96	26.21	26.16	36.30	59.81	61.15	61.89
493.90	Asthma, unspecified type, unspecified exacerbation status (%)	27.50	5.09	4.58	4.32	24.78	9.11	8.24	7.41	25.09	2.72	2.41	2.23	31.61	5.77	5.24	5.02	20.88	6.36	6.09	5.87
493.91	Asthma, unspecified type, with status asthmaticus (%)	4.59	21.76	23.48	23.50	5.62	35.21	37.55	38.40	7.30	46.64	48.54	48.46	1.64	5.45	5.42	5.33	0.73	1.23	1.11	1.05
493.92	Asthma, unspecified type, with (acute) exacerbation (%)	44.79	36.09	34.86	34.74	25.78	23.45	22.59	21.02	39.64	20.65	18.98	17.86	56.83	54.26	54.52	55.33	34.66	24.79	24.38	24.60
786.07	Wheezing (%)	3.85	1.42	1.26	1.27	16.68	8.77	7.45	7.54	3.72	1.76	1.55	1.57	0.73	0.04	0.03	0.03	1.12	0.15	0.13	0.14
Pneumonia (Overall N)		***98140***	***59681***	***51981***	***49489***	***11178***	***2891***	***2501***	***2327***	***19455***	***4513***	***3818***	***3304***	***33193***	***18827***	***16072***	***15143***	***34313***	***33449***	***29589***	***28714***
486	Pneumonia, organism unspecified (%)	90.31	85.17	85.30	85.87	87.52	71.01	71.69	72.15	91.47	80.19	79.60	82.42	92.16	86.26	86.29	86.59	88.77	86.45	86.64	86.99
480.1	Pneumonia due to respiratory syncytial virus (%)	0.64	1.00	0.95	0.88	4.31	14.84	14.15	13.67	0.68	3.15	3.20	3.18	0.02	0.08	0.05	0.04	0.02	0.03	0.03	0.03
480.9	Viral pneumonia, unspecified (%)	0.96	1.15	1.12	1.07	3.23	7.71	7.44	7.56	1.50	3.28	3.35	3.57	0.51	0.90	0.94	0.85	0.34	0.43	0.39	0.38
CVD (Overall N)		***210250***	***248810***	***176059***	***155089***	***406***	***196***	***153***	***125***	***1054***	***393***	***294***	***229***	***80895***	***90262***	***61416***	***53021***	***12789***	***15795***	***11419***	***101713***
410.71	Subendocardial infarction, initial episode of care (%)	8.13	7.86	10.13	10.90	0.00	0.00	0.00	0.00	0.19	0.76	1.02	0.87	6.77	7.13	9.38	10.14	9.08	8.30	10.57	11.34
414.01	Coronary atherosclerosis, of native coronary artery (%)	10.36	21.18	14.48	11.65	0.00	0.00	0.00	0.00	0.00	0.00	0.00	0.00	13.20	27.40	19.95	16.36	8.68	17.71	11.60	9.24
427.0	Paroxysmal supraventricular tachycardia (%)	1.49	0.62	0.75	0.80	2.96	9.69	9.15	8.00	6.07	2.80	3.40	4.37	2.55	0.84	1.02	1.11	0.78	0.48	0.59	0.61
427.31	Atrial fibrillation (%)	6.99	6.11	6.67	6.64	0.00	0.00	0.00	0.00	3.13	6.87	8.16	9.17	6.68	5.64	6.39	6.54	7.24	6.38	6.83	6.69
427.5	Cardiac arrest (%)	3.66	0.16	0.21	0.22	43.35	7.14	8.50	10.40	14.23	2.54	2.72	3.49	4.14	0.16	0.22	0.24	3.14	0.14	0.19	0.20
427.89	Other specified cardiac dysrhythmias, other (%)	4.14	2.20	2.49	2.66	22.17	21.43	25.49	28.80	40.32	24.94	17.69	18.34	5.05	1.97	2.23	2.40	3.21	2.25	2.57	2.72
427.9	Cardiac dysrhythmia, unspecified (%)	0.36	0.07	0.09	0.09	2.22	0.00	0.00	0.00	5.50	1.53	1.70	1.31	0.46	0.07	0.09	0.09	0.24	0.07	0.09	0.09
428.0	Congestive heart failure, unspecified type (%)	23.30	21.11	24.72	26.07	17.00	47.96	43.79	42.40	3.98	13.74	14.63	9.61	17.72	16.26	19.13	20.53	27.01	23.87	27.72	28.97
434.91	Cerebral artery occlusion, unspecified, with cerebral infarction (%)	6.92	6.17	8.02	8.93	1.23	3.06	3.92	4.00	1.33	3.05	4.08	5.24	5.49	5.10	6.86	7.82	7.89	6.79	8.66	9.52
435.9	Unspecified transient cerebral ischemia (%)	5.83	3.08	4.02	4.51	0.00	0.00	0.00	0.00	0.66	1.02	1.36	1.75	4.78	2.62	3.54	4.03	6.56	3.35	4.29	4.77
443.89	Other specified peripheral vascular diseases (%)	0.05	0.05	0.02	0.02	5.91	0.51	0.65	0.80	0.28	0.00	0.00	0.00	0.04	0.06	0.02	0.02	0.03	0.05	0.02	0.02
453.8	Venous embolism and thrombosis of other specified veins (%)	1.91	2.13	1.88	1.62	0.99	2.04	1.31	0.80	3.61	7.89	7.82	8.30	2.69	2.95	2.67	2.40	1.40	1.64	1.43	1.21
Dysrhythmia (Overall N)		***43449***	***33449***	***25183***	***22261***	***290***	***80***	***70***	***59***	***792***	***195***	***131***	***106***	***18467***	***10810***	***8094***	***7206***	***23897***	***22364***	***16888***	***14890***
427.0	Paroxysmal supraventricular tachycardia (%)	7.22	4.63	5.27	5.55	4.14	23.75	20.00	16.95	8.08	5.64	7.63	9.43	11.18	7.01	7.76	8.19	4.17	3.41	3.99	4.20
427.1	Paroxysmal ventricular tachycardia (%)	3.98	8.14	6.54	6.43	0.00	0.00	0.00	0.00	1.14	5.13	5.34	3.77	4.04	10.16	8.08	8.03	4.08	7.22	5.84	5.70
427.31	Atrial fibrillation (%)	33.85	45.48	46.69	46.26	0.00	0.00	0.00	0.00	4.17	13.85	18.32	19.81	29.28	47.17	48.54	48.11	38.77	45.10	46.22	45.74
427.32	Atrial flutter (%)	4.03	6.36	6.27	6.08	0.00	1.25	1.43	0.00	1.64	9.74	9.92	8.49	4.04	7.81	7.56	7.27	4.16	5.65	5.64	5.51
427.5	Cardiac arrest (%)	17.74	1.17	1.47	1.56	60.69	17.50	18.57	22.03	19.07	5.13	6.11	7.55	18.18	1.34	1.64	1.76	16.82	1.00	1.27	1.34
427.81	Sinoatrial node dysfunction (%)	6.16	14.40	12.55	11.73	0.00	0.00	0.00	0.00	0.38	3.08	3.05	1.89	1.64	5.25	4.46	4.01	9.92	18.98	16.55	15.59
427.89	Other specified cardiac dysrhythmias, other (%)	20.03	16.35	17.45	18.51	31.03	52.50	55.71	61.02	53.66	50.26	39.69	39.62	22.13	16.43	16.90	17.62	17.17	15.89	17.39	18.62
427.9	Cardiac dysrhythmia, unspecified (%)	1.73	0.54	0.63	0.65	3.10	0.00	0.00	0.00	7.32	3.08	3.82	2.83	2.03	0.59	0.65	0.64	1.29	0.50	0.59	0.64
CHF	***Overall N***	***52989***	***57426***	***47668***	***44337***	***72***	***96***	***68***	***54***	***45***	***56***	***44***	***23***	***15432***	***16018***	***12869***	***11936***	***37440***	***41256***	***34687***	***32324***
428.0	Congestive heart failure, unspecified type (%)	92.56	91.52	91.34	91.24	95.83	97.92	98.53	98.15	93.33	96.43	97.73	95.65	93.06	91.70	91.35	91.27	92.35	91.43	91.32	91.21

*The table lists only ICD codes representing at least 5% of the case group in any of the data sets overall or in any of the age groups. Percentages do not add to 100 due to ICD codes representing <5% of the case group in all data sets not being listed.

ICD-9: International Classification of Diseases, 9th revision, Clinical Modification, RD: respiratory disease group, CVD: cardiovascular disease group, CHF: congestive heart failure, ED: emergency department, HA: hospital admission
