# Supplementary material for: Comparison of emergency department and hospital admissions data for air pollution time-series studies
Source: Environ Health. 2012 Sep 21;11:70. doi: 10.1186/1476-069X-11-70 (PMC3511882; doi:10.1186/1476-069X-11-70)
Supplement: Additional file 3 — Table S3. Summary (lags 0–4) distributed lag model risk ratios for each data set for Ozone, Tudor Street monitor, RR expressed per IQR (27.3 ppb) increment. Description: Table of results of overall time series models for the associations between daily counts of visits for the selected outcomes and daily ozone concentrations. [file 1476-069X-11-70-S3.pdf]

Table A.3- Summary (lags 0-4) distributed lag model risk ratios for each data set for Ozone, Tudor Street monitor, RR expressed per IQR (27.3 ppb) increment

		ED				All HA				Non-Elective HA				HA through ED			
Outcome	Sub-Group	RR	Lower 95% CI	Upper 95% CI	p-value	RR	Lower 95% CI	Upper 95% CI	p-value	RR	Lower 95% CI	Upper 95% CI	p-value	RR	Lower 95% CI	Upper 95% CI	p-value
RD	All	1.020	0.999	1.043	0.066	1.003	0.967	1.039	0.882	1.007	0.969	1.046	0.732	1.004	0.965	1.045	0.833
	Age 0-1 years	1.049	1.006	1.094	0.024	1.027	0.907	1.163	0.676	1.057	0.927	1.206	0.406	1.086	0.947	1.246	0.238
	Age 2-18 years	1.038	1.002	1.075	0.040	1.101	0.995	1.218	0.062	1.110	0.998	1.233	0.054	1.119	1.001	1.249	0.047
	Age 19-64 years	1.011	0.984	1.039	0.444	0.982	0.926	1.042	0.556	0.995	0.934	1.061	0.889	0.996	0.934	1.063	0.909
	Age 65+ years	0.977	0.933	1.023	0.315	0.993	0.944	1.044	0.775	0.984	0.933	1.038	0.556	0.973	0.921	1.028	0.330
	Zip code not in poverty area	1.019	0.996	1.043	0.112	1.001	0.962	1.042	0.962	1.007	0.965	1.050	0.757	1.007	0.964	1.053	0.754
	Zip code in poverty area	1.026	0.991	1.062	0.141	1.012	0.940	1.088	0.758	1.011	0.935	1.093	0.785	1.000	0.924	1.081	0.995
Asthma/Wheeze	All	1.069	1.028	1.111	0.001	1.070	0.992	1.154	0.082	1.101	1.017	1.192	0.018	1.106	1.020	1.200	0.014
	Age 0-1 years	1.093	0.992	1.204	0.072												
	Age 2-18 years	1.116	1.053	1.182	0.000	1.175	1.028	1.344	0.018	1.175	1.025	1.347	0.021	1.180	1.026	1.357	0.020
	Age 19-64 years	1.012	0.960	1.068	0.650	0.993	0.886	1.113	0.900	1.026	0.908	1.159	0.680	1.010	0.891	1.145	0.876
	Age 65+ years	1.100	0.932	1.298	0.261	1.139	0.922	1.406	0.228					1.244	0.985	1.572	0.067
	Zip code not in poverty area	1.066	1.019	1.116	0.006	1.054	0.960	1.157	0.268	1.091	0.989	1.205	0.083	1.108	1.001	1.227	0.048
	Zip code in poverty area	1.076	1.015	1.141	0.014	1.108	0.974	1.260	0.119	1.124	0.983	1.285	0.087	1.108	0.969	1.267	0.135
Pneumonia	All	1.009	0.971	1.049	0.652	1.008	0.962	1.056	0.746	0.998	0.949	1.050	0.951	0.997	0.947	1.050	0.916
	Age 0-1 years	1.076	0.960	1.207	0.208	1.309	1.048	1.636	0.018	1.341	1.056	1.703	0.016	1.374	1.071	1.763	0.012
	Age 2-18 years	0.989	0.905	1.080	0.804	0.943	0.797	1.117	0.497	0.928	0.774	1.112	0.417	0.948	0.780	1.153	0.596
	Age 19-64 years	1.035	0.972	1.102	0.280	0.972	0.895	1.056	0.506	0.989	0.904	1.082	0.813	0.985	0.899	1.079	0.739
	Age 65+ years	0.978	0.920	1.041	0.487	1.017	0.957	1.080	0.588	0.990	0.928	1.056	0.763	0.987	0.924	1.055	0.703
	Zip code not in poverty area	1.004	0.963	1.047	0.850	1.005	0.956	1.058	0.835	0.998	0.945	1.054	0.930	0.997	0.943	1.055	0.924
	Zip code in poverty area	1.035	0.947	1.131	0.447	1.018	0.910	1.139	0.755	1.003	0.888	1.134	0.960	1.002	0.886	1.132	0.980
CVD	All	1.002	0.979	1.026	0.847	0.990	0.966	1.015	0.444	1.004	0.978	1.031	0.744	1.002	0.975	1.030	0.896
	Age 0-1 years																
	Age 2-18 years																
	Age 19-64 years	0.980	0.945	1.017	0.287	0.968	0.932	1.005	0.086	0.980	0.939	1.023	0.360	0.965	0.922	1.010	0.122
	Age 65+ Years	1.016	0.987	1.047	0.286	1.004	0.975	1.033	0.808	1.018	0.986	1.051	0.268	1.023	0.990	1.057	0.180
	Zip code not in poverty area	0.991	0.966	1.016	0.470	0.983	0.957	1.009	0.198	0.995	0.967	1.024	0.747	0.991	0.962	1.020	0.533
	Zip code in poverty area	1.065	1.007	1.126	0.027	1.036	0.979	1.096	0.222	1.057	0.991	1.128	0.092	1.061	0.994	1.132	0.073
Dysrhythmia	All	1.003	0.953	1.055	0.918	0.995	0.939	1.055	0.871	1.008	0.945	1.076	0.799	1.004	0.936	1.077	0.913
	Age 0-1 years																
	Age 2-18 years																
	Age 19-64 years	1.006	0.932	1.085	0.886	0.936	0.847	1.035	0.200	0.993	0.886	1.113	0.903	0.992	0.880	1.119	0.898
	Age 65+ years	0.999	0.933	1.070	0.978	1.026	0.957	1.100	0.473	1.016	0.939	1.100	0.687	1.011	0.928	1.100	0.805
	Zip code not in poverty area	0.981	0.929	1.036	0.494	0.995	0.936	1.058	0.876	1.004	0.938	1.076	0.902	0.993	0.922	1.070	0.854
	Zip code in poverty area	1.145	1.008	1.299	0.037	1.000	0.850	1.177	0.999	1.048	0.874	1.257	0.615	1.083	0.898	1.306	0.405
CHF	All	1.068	1.019	1.120	0.006	1.069	1.022	1.118	0.004	1.076	1.025	1.130	0.003	1.076	1.023	1.133	0.005
	Age 0-1 years																
	Age 2-18 years																
	Age 19-64 years	1.046	0.961	1.139	0.299	1.074	0.988	1.166	0.093	1.072	0.978	1.176	0.137	1.058	0.961	1.165	0.254
	Age 65+ years	1.079	1.021	1.140	0.007	1.068	1.013	1.126	0.014	1.078	1.019	1.141	0.009	1.084	1.022	1.150	0.008
	Zip code not in poverty area	1.058	1.004	1.114	0.035	1.059	1.006	1.114	0.028	1.054	0.998	1.114	0.058	1.056	0.997	1.118	0.062
	Zip code in poverty area	1.105	1.004	1.217	0.040	1.107	1.006	1.218	0.037	1.163	1.047	1.291	0.005	1.152	1.037	1.280	0.009

RD: respiratory disease group, CVD: cardiovascular disease group, CHF: congestive heart failure, RR: risk ratio, 95% CI: 95% confidence interval, IQR: interquartile range, ED: emergency department, HA: hospital admission
