# Supplementary material for: Comparison of emergency department and hospital admissions data for air pollution time-series studies
Source: Environ Health. 2012 Sep 21;11:70. doi: 10.1186/1476-069X-11-70 (PMC3511882; doi:10.1186/1476-069X-11-70)
Supplement: Additional file 4 — Table S4. Summary (lags 0–4) distributed lag model risk ratios for each data set for PM2.5, Blair Street Monitor, RR expressed per IQR (9.3 μg/m3) increment. Description: Table of results of overall time series models for the associations between daily counts of visits for the selected outcomes and daily PM2.5 concentrations. [file 1476-069X-11-70-S4.pdf]

Table A.4-Summary (lags 0-4) distributed lag model risk ratios for each data set for $PM_{2.5}$, Blair Street Monitor, RR expressed per IQR (9.3 µg/m³) increment

		ED				All HA				Non-Elective HA				HA through ED			
Outcome	Sub-Group	RR	Lower 95% CI	Upper 95% CI	p-value	RR	Lower 95% CI	Upper 95% CI	p-value	RR	Lower 95% CI	Upper 95% CI	p-value	RR	Lower 95% CI	Upper 95% CI	p-value
RD	All	1.012	1.002	1.022	0.024	1.001	0.984	1.018	0.922	0.998	0.980	1.017	0.855	1.004	0.986	1.023	0.659
	Age 0-1 years	1.018	0.999	1.037	0.061	1.027	0.973	1.084	0.340	1.018	0.961	1.079	0.538	1.033	0.973	1.097	0.286
	Age 2-18 years	1.032	1.015	1.049	0.000	1.014	0.966	1.064	0.566	1.014	0.963	1.066	0.603	1.029	0.976	1.086	0.289
	Age 19-64 years	1.000	0.987	1.013	0.997	0.996	0.969	1.025	0.801	0.994	0.964	1.024	0.685	1.004	0.974	1.035	0.789
	Age 65+ years	0.999	0.978	1.021	0.933	0.997	0.974	1.021	0.805	0.995	0.970	1.021	0.717	0.995	0.969	1.021	0.692
	Zip code not in poverty area	1.013	1.002	1.025	0.016	1.000	0.982	1.019	0.974	1.000	0.980	1.020	0.974	1.003	0.982	1.024	0.791
	Zip code in poverty area	1.006	0.990	1.022	0.487	1.006	0.971	1.041	0.757	0.995	0.958	1.033	0.801	1.012	0.974	1.050	0.544
Asthma/Wheeze	All	1.027	1.007	1.046	0.007	1.032	0.995	1.072	0.093	1.029	0.990	1.070	0.151	1.029	0.989	1.071	0.155
	Age 0-1 years	1.047	0.999	1.097	0.057												
	Age 2-18 years	1.050	1.021	1.080	0.001	1.052	0.985	1.123	0.132	1.039	0.972	1.112	0.262	1.042	0.973	1.117	0.241
	Age 19-64 years	1.002	0.976	1.029	0.871	1.029	0.974	1.087	0.307	1.019	0.961	1.081	0.525	1.033	0.972	1.097	0.294
	Age 65+ years	0.998	0.922	1.079	0.951	0.960	0.869	1.061	0.425					0.981	0.879	1.094	0.731
	Zip code not in poverty area	1.022	0.999	1.045	0.056	1.039	0.993	1.087	0.101	1.041	0.992	1.092	0.100	1.038	0.988	1.091	0.135
	Zip code in poverty area	1.036	1.006	1.066	0.017	1.020	0.957	1.086	0.550	1.006	0.941	1.075	0.861	1.013	0.948	1.082	0.702
Pneumonia	All	0.990	0.973	1.008	0.284	0.972	0.951	0.994	0.011	0.966	0.943	0.989	0.005	0.975	0.951	0.999	0.041
	Age 0-1 years	0.964	0.916	1.014	0.158	0.936	0.846	1.034	0.193	0.927	0.832	1.033	0.170	0.979	0.875	1.095	0.712
	Age 2-18 years	1.035	0.995	1.077	0.086	0.991	0.918	1.069	0.809	1.001	0.921	1.088	0.982	1.025	0.938	1.120	0.585
	Age 19-64 years	0.989	0.960	1.018	0.439	0.963	0.926	1.002	0.060	0.962	0.922	1.004	0.078	0.976	0.935	1.019	0.265
	Age 65+ years	0.975	0.947	1.004	0.095	0.978	0.950	1.006	0.128	0.967	0.938	0.997	0.034	0.969	0.939	1.000	0.050
	Zip code not in poverty area	0.990	0.970	1.009	0.296	0.970	0.947	0.993	0.012	0.965	0.941	0.991	0.007	0.970	0.944	0.996	0.023
	Zip code in poverty area	0.993	0.953	1.034	0.727	0.987	0.937	1.040	0.620	0.972	0.918	1.029	0.331	1.004	0.948	1.063	0.896
CVD	All	1.010	0.998	1.022	0.103	1.000	0.988	1.013	0.951	1.002	0.989	1.015	0.813	1.004	0.991	1.018	0.542
	Age 0-1 years																
	Age 2-18 years																
	Age 19-64 years	1.010	0.992	1.028	0.295	1.003	0.985	1.022	0.736	1.001	0.980	1.023	0.903	1.005	0.983	1.028	0.660
	Age 65+ Years	1.011	0.996	1.025	0.157	0.999	0.985	1.014	0.939	1.002	0.987	1.018	0.760	1.004	0.988	1.021	0.592
	Zip code not in poverty area	1.011	0.999	1.024	0.084	1.002	0.989	1.015	0.763	1.004	0.990	1.018	0.620	1.006	0.991	1.020	0.430
	Zip code in poverty area	1.002	0.975	1.031	0.861	0.990	0.962	1.018	0.468	0.990	0.958	1.022	0.525	0.995	0.964	1.027	0.759
Dysrhythmia	All	1.020	0.995	1.046	0.123	1.006	0.978	1.036	0.672	1.013	0.981	1.046	0.435	1.027	0.992	1.063	0.134
	Age 0-1 years																
	Age 2-18 years																
	Age 19-64 years	1.023	0.986	1.062	0.231	0.995	0.946	1.046	0.843	1.007	0.952	1.066	0.807	1.018	0.959	1.080	0.561
	Age 65+ years	1.020	0.986	1.055	0.249	1.015	0.980	1.050	0.405	1.017	0.979	1.057	0.384	1.030	0.989	1.074	0.155
	Zip code not in poverty area	1.018	0.991	1.046	0.190	1.011	0.981	1.043	0.466	1.018	0.985	1.053	0.289	1.027	0.991	1.065	0.148
	Zip code in poverty area	1.029	0.966	1.095	0.378	0.968	0.893	1.050	0.435	0.970	0.887	1.062	0.515	1.020	0.930	1.119	0.668
CHF	All	1.009	0.987	1.033	0.419	1.008	0.986	1.031	0.483	1.008	0.984	1.032	0.538	1.010	0.986	1.036	0.414
	Age 0-1 years																
	Age 2-18 years																
	Age 19-64 years	0.980	0.940	1.021	0.333	1.006	0.966	1.048	0.759	0.992	0.948	1.038	0.735	0.998	0.952	1.047	0.949
	Age 65+ years	1.023	0.997	1.051	0.088	1.010	0.984	1.036	0.462	1.015	0.987	1.043	0.306	1.016	0.987	1.046	0.270
	Zip code not in poverty area	1.003	0.978	1.029	0.812	1.001	0.976	1.026	0.960	1.002	0.976	1.030	0.861	1.005	0.977	1.033	0.737
	Zip code in poverty area	1.030	0.983	1.080	0.208	1.035	0.988	1.085	0.146	1.026	0.975	1.080	0.325	1.029	0.977	1.083	0.283

RD: respiratory disease group, CVD: cardiovascular disease group, CHF: congestive heart failure, RR: risk ratio, 95% CI: 95% confidence interval, IQR: interquartile range, PM2.5: particulate matter ≤2.5 micrometers in diameter, ED: emergency department, HA: hospital admission
